# Clinical practice recommendations for the care of infants with stage 5 chronic kidney disease (CKD5)

**DOI:** 10.1007/s00467-012-2300-z

**Published:** 2012-10-09

**Authors:** Aleksandra M. Zurowska, Michel Fischbach, Alan R. Watson, Alberto Edefonti, Constantinos J. Stefanidis, on behalf of the European Paediatric Dialysis Working Group

**Affiliations:** 1 Department Paediatric & Adolescent Nephrology & Hypertension, Medical University of Gdansk, Ul. Debinki 7, 80-211 Gdansk, Poland; 2 Hopital de Hautepierre, Strasbourg, France; 3 Nottingham University Hospitals NHS Trust, Nottingham, UK; 4 Clinica Pediatrica De Marchi, Fondazione Ca’ Granda IRCCS Ospedale Maggiore Policlinico, Milan, Italy; 5 “A & P Kyriakou” Children’s Hospital, Athens, Greece

**Keywords:** Infants, Neonates, Chronic kidney disease (CKD), Haemodialysis (HD), Peritoneal dialysis (PD)

## Abstract

**Background:**

To provide recommendations for the care of infants with stage 5 chronic kidney disease (CKD5).

**Setting:**

European Paediatric Dialysis Working Group.

**Data Sources:**

Literature on clinical studies involving infants with CKD5 (end stage renal failure) and consensus discussions within the group.

**Recommendations:**

There has been an important change in attitudes towards offering RRT (renal replacement therapy) to both newborns and infants as data have accumulated on their improved survival and long-term outcomes. The management of this challenging group of patients differs in a number of ways from that of older children. The authors have summarised the basic recommendations for treating infants with CKD5 in order to support the multidisciplinary teams who endeavour on this difficult task.

## Introduction

The European Paediatric Dialysis Working Group (EPDWG) was established in 1999 and comprises of paediatric nephrologists with a major interest in dialysis from 13 European countries. Recommendations produced by the EPDWG have been endorsed by the European Society of Paediatric Nephrology.

The number of children initiating dialysis in the first year of life has increased over the last decades. According to international registries infants constitute 11 % of all children initiating renal replacement therapy (RRT) [1, 2]. In Europe the incidence of end stage renal disease (ESRD) in children <1 year of age is between 9 and 16 per million age-related population per year, which is nearly double the figure for the whole paediatric age group (0–15 years) [1, 3]. In regions where dialysis is not routinely available for neonates or infants, the reported number of children initiating RRT in this age group is lower. The majority of children requiring RRT in infancy have congenital anomalies of the kidney and urinary tract (CAKUT) or hereditary diseases [congenital nephrotic syndrome (CNS), autosomal recessive polycystic kidney disease (ARPKD)] which are frequently recognised on prenatal ultrasound images. Parents need to be fully informed at this stage on the possibilities of treatment and survival rates for their newborn. With contemporary management, the outcome of both congenital and acquired diseases (cortical necrosis, renal vein thrombosis, hemolytic uremic syndrome) requiring RRT in infancy has greatly improved although it is still inferior to that of older children [4–10]. Neonates have outcomes comparable to those in later infancy [2, 8]. An important cause of death in this age group is due to non-renal co-morbidities. Prompt recognition of all factors which will have an impact on further survival is a basic issue in the assessment of infants with ESRD.

Due to the relatively small number of infant patients seen at single centers, data from randomised trials are not available and the recommendations presented here are based on literature research (Medline), abstracts presented at international conferences, consensus meetings of the EPDWG and extended e-mail discussion. The grading of the recommendations is based on National Kidney Foundation Disease Outcomes Quality Initiative (KDOQI) nomenclature and methods [11]. Evidence level 1 indicates a recommendation that most experts would want to be the course of action in most patients. Evidence level 2 indicates a suggestion that the majority of experts would want to be realised, but some would not, and that different choices will be appropriate in different patients, in accordance with the patients’ values and preferences. Level A indicates high-quality scientific evidence, B moderate, C low and D very low, implying that the true effect will be close (A) or often far (D) from the estimate.

### Recommendation 1: Counselling for families of infants with antenatally or postnatally diagnosed kidney disease must involve the paediatric nephrology team who will be providing long term care (1B).

The majority of children with stage 5 chronic kidney disease (CKD5) in the first year of life have CAKUT (renal aplasias, dysplasias, hypoplasias and obstructive uropathies). Among boys, posterior urethral valve (PUV) and Prune Belly syndrome predominate, and in girls, the VATER (vertebral–anal–tracheoesophageal–renal) sequence. The structural defects can be isolated or part of a syndrome (e.g. Alagille, Jeune syndromes). Co-morbidities are frequently present in both isolated or syndromic CAKUT and hereditary diseases [12, 13]. These include cardiac defects, liver and gut disease, central nervous system involvement, ear, ocular and osseous defects. It is particularly difficult to give a firm prognosis for antenatally detected urinary tract abnormalities such as PUV as mortality often depends upon the degree of oligohydramnios and subsequent lung function. Prognosis should always be guarded and continuity of support from an experienced paediatric nephrologist as well as urologist is suggested under such circumstances.

Co-morbidities may be more frequently present in the group with CAKUT than in infants with acquired causes of CKD, with the former accounting for 28 % of infants in the experience of the Italian Registry of Paediatric Chronic Dialysis [14]. Many of these children will need a multiprofessional team approach (neonatologist, nephrologist, urologist, gastroenterologist, cardiologist, neurologist, dietitian, speech and play therapists, psychologist) and will likely require surgical interventions during early childhood. Enormous dedication is required from their caretakers, who will need training and psychosocial support [15].

Contemporary management of these complex medical problems has led to a significant improvement in the survival rates of infants with CKD5. The outcome of diseases previously looked upon as fatal (e.g. CNS, severe ARPKD) has significantly improved [16, 17]. Although the mortality rate for babies starting dialysis at <1 month of age is higher than that for older infants, the proportion who recover renal function is greater [2]. The British Association of Paediatric Nephrology (BAPN), North American Pediatric Renal Trials and Collaborative Studies (NAPRTCS) and Italian Registry data confirm that neonates can initiate long-term dialysis with outcomes comparable to those achieved in later infancy [2, 5, 14]. A major factor influencing the final outcome of infants with CKD5 is existing co-morbidities [9, 10, 12, 14, 18]. Their prompt recognition and assessment of their impact on further survival are important issues in the decision for commencing or withholding RRT.

### Recommendation 2: Consensus decision-making based on guidelines is recommended for both commencement and withholding of RRT in the neonate or infant (1B).

The attitudes of both primary and specialty care providers towards the treatment of neonates and infants with ESRD have evolved over the last decades. RRT is increasingly looked upon as a feasible option for this age group and initiating dialysis in infancy has become a standard practice in developed countries. Nevertheless, nearly half of the decisions on withholding or withdrawing life-supporting therapy concern children <1 year of age [19]. Paediatric nephrology centers advocate multiprofessional team discussions, which may be guided by a clinical ethics committee where appropriate, to decide upon offering RRT to newborns and infants [20, 21]. The principle criterion used to make these decisions is the best interest of the child. The following issues should be addressed for discussion with members of the medical and paramedical team:

1. Short and long-term prognosis (co-morbidities influencing medical care and outcome).
2. Medical care issues (availability of equipment, expertise and financial resources, possibility of future transplantation).
3. The predicted quality of life for the child (additional disabilities) and the family. This difficult issue is associated with the feasibility of delivering high-dependency care for the infant by the caretakers, involving their social situation and family structure and possible sources of support.

The parents need to obtain detailed information on all issues and be actively involved in the decision-making process. Withholding treatment requires careful counselling as the outcome can be difficult to assess, especially in neonates who may improve and come off dialysis. Conservative management may last for several months, and the parents may bond with the child and change their opinion; by this time irreversible growth failure and developmental damage may have occurred [22]. The decision on offering RRT to the infant should be made as soon as possible as it will initiate immediate intensive conservative management aimed at attaining normal development and appropriate planning of necessary surgical interventions prior to dialysis or transplantation [23].

### Recommendation 3: The timing of initiating RRT in infants with CKD5 needs to be individualised with a major determinant being maintenance of adequate growth, nutrition and development (1B).

European guidelines recommend starting dialysis in children when the estimated glomerular filtration rate (eGFR) is between 15 and 10 ml/min/1.73 m^2^—unless the child is growing well and is asymptomatic [24]. Infants with high urine output may thrive despite low eGFR [25]. In infants, eGFR is a weaker determinant for initiating dialysis. Creatinine- and cystatin C-based estimations are less accurate and often not easy to interpret. The eGFR of infants with CKD will increase even up to 3 years of age [26]. Children with renal dysplasia who manage to achieve a eGFR of >15 ml/min/1.73 m^2^ by 6 months of age will probably not need RRT for many months or years [27]. Ultimately, 4 % of infants and 15 % of neonates may recover renal function and terminate dialysis [1, 2]. On the other hand, infants with CNS or ARPKD may require dialysis at a relatively high eGFR due to elective nephrectomy in preparation for transplantation in the former and nephrectomy due to malignant hypertension or complications of the nephromegaly in the latter. An important determinant of initiating dialysis in infants is the maintenance of satisfactory growth and development. Dialysis may be necessary to achieve adequate nutrition, necessary control of metabolic acidosis, electrolyte balance and blood pressure. Persistent oliguria in infants will require an early start of dialysis to prevent fluid overload due to their predominantly liquid diet [28].

### Recommendation 4: Peritoneal dialysis involves a multidisciplinary care approach and is the preferred modality of renal replacement therapy in infants with CKD5 (1B). 

Renal replacement therapy in infants is one of the most challenging and demanding treatments in nephrology. An integrated care modality should be considered and individualised for each child. Transplantation is looked upon as the ultimate goal but will be postponed in most centres until the infant reaches a body weight of 8–10 kg [29]. Peritoneal dialysis (PD) is the method of choice for initiating RRT in the youngest age group and has been shown to preserve residual renal function which in turn improves the clearance of larger molecules and enables provision of larger feeding volumes. PD enables preservation of vascular access for future use. Haemodialysis (HD) in infants and small children is an effective and safe form of RRT and must be available as an option, but problems with maintaining vascular access limit its long-term use [30–32]. HD is chosen as the initial mode of RRT in infants with primary oxalosis and those with contraindications for PD (omphalocele, gastroschisis, diaphragmatic hernia, obliterated peritoneal cavity, bladder exstrophy). Short-term HD may also be necessary following the insertion or replacement of PD catheters and abdominal surgery.

Infant PD requires good dialysis access, as well as the appropriate tubing and cyclers to allow low fill volumes. Automated PD is the preferred mode of dialysis and can be performed in infants with fill volumes greater than 100 ml using paediatric tubing and standard cyclers with paediatric software. The use of smaller fill volumes (< 50–60 ml) may lead to several pitfalls:

1. Frequent alarms due to slow outflow or low flow rates that are not detected by the cycler or negative ultrafiltration observed in polyuric infants who reabsorb water and sodium from the dialysate.
2. Relatively large recirculation volume (17–45 ml) may lead to a poor clearance of uremic toxins.

For infants weighing less than 2–3 kg with fill volumes of <100 ml short-term dialysis needs to be performed manually with the use of commercially available two chamber sets with a controlled temperature. These allow inflow and outflow volumes to be measured with an accuracy of 1 ml. Manual exchanges in infants are easily performed in a hospital setting, and infants are seldom discharged home until larger fill volumes and therefore automated PD (APD) is achieved [33].

### Recommendation 5: Surgical catheter insertion and partial omentectomy is recommended for infants. Exit site should be far from the diaper area and from any ostomies. Catheters need to be immobilised to prevent tearing and pulling and dialysis postponed for 2–3 weeks if possible allowing healing (1C).

Paediatric-sized catheters are used for infants up to 10 kg. Although no evidence exists on the influence of the type of catheters used in infants on the infectious and non-infectious complication rates, most centres use single cuff catheters for infants of <3 kg and double cuff catheters for infants weighing 3–10 kg (external cuff >2 cm from exit site).

Both straight and coiled catheters are used, although the latter are preferred as they are shorter and catheter dislodging has been shown to be less frequent in older children [24]. Catheters should be implanted by a dedicated surgeon during a surgical procedure, though laparoscopic placement has also been described in this age group. Partial omentectomy is recommended due to the higher risk of catheter obstruction in infants, but there is as yet no evidence of its prophylactic efficacy [24, 34]. A para-median or lateral entry into the peritoneum is performed with fixation of the internal cuff in the rectus muscle. The exit site is lateral or sometimes at the level of the umbilicus so as to be outside the diaper area and at a safe distance from any ostomies on the abdominal wall. Presternal exit sites are used in some centres [35]. Immobilisation of the catheter by dressing is advocated to allow healing and to prevent pulling and tearing. Due to the high risk of fluid leaks and hernias in infants, a delay of 2–3 weeks in dialysis following catheter implantation is beneficial. Fibrin glue at the internal cuff has been shown to decrease leaking complications [36]. Elective herniotomy should be considered on the contralateral site if there is evidence of inguinal or other hernias. Catheter placement and the treatment of complications require the input of a dedicated paediatric surgeon; the importance of this specialist cannot be overestimated, as infants have a potentially high incidence of catheter complications and face a lifetime of renal replacement therapy [31]. Many infants will also need careful planning of urologic procedures to prepare the urinary tract for transplantation [37, 38].

### Recommendation 6: The dialysis prescription in the infant must be individualised and adapted to the infant’s urine output and nutritional requirements (1B)

Infant dialysis will require lower fill volumes (600–900 ml/m^2^), shorter dwell times (30–40 min), more frequent exchanges (12–16) and longer duration of dialysis (10–16 h) than older children [33, 39]. Infants tolerate lower volumes of dialysis fluid than older children, and fill volumes need to be restricted to 600–900 ml/m^2^. The last (daytime) fill volume should be only one-half the night fill volume. High fill volumes increase the risk of leakage, hernias and gastro-oesophageal reflux and may compromise respiration. Increasing the fill volume over time may result in better ultrafiltration (UF) and clearance. Intraperitoneal pressure (IPP) is a helpful guide to assess the amount of fill volume tolerated in infants and should ideally be kept at <10 cm H_2_O [40]. Low fill volumes are safer for infants but result in poor clearance and low UF and induce a hyper-permeable exchange [41]. When ultrafiltration is low, there is an additional risk of dialysis recirculation in the tubing (35–45 ml). Increases in the UF volume and a better clearance can be achieved by shortening dwell times to 1 h or less (30–45 min), increasing the number of exchanges to 12–16 or increasing the glucose concentration in the dialysis fluid. In the anuric infant, despite the long duration of dialysis (10–16 h), clearance may still not be sufficient. In polyuric infants, dialysate absorption is often seen and can be sometimes beneficial. Additional daytime exchanges may increase both creatinine and phosphate clearances [42–47].

### Recommendation 7: Infants should receive biocompatible fluids to allow for long-term preservation of the peritoneal membrane function (1B).

There are no available studies in the infant population comparing the use of different dialysis fluids. It seems reasonable to deliver the most biocompatible fluid available with neutral pH and low levels of glucose degradation products to minimise the adverse effects of dialysis fluids on the infant peritoneal membrane [48, 49]. Fluids with neutral pH preserve membrane function, and children show lower IPP [50]. Frequent cycles can result in alkalosis and lower buffer content dialysate is then recommended. Fluids with the lowest possible glucose concentration should be used, but polyuric infants may absorb fluid from low glucose exchanges, whereas anuric children may require higher glucose fluids for adequate UF [49, 51]. Calcium concentration (1.25 or 1.75 mEq/l) needs to be individualised to provide sufficient calcium for growth and achieve target calcium, phosphorus and parathyroid hormone values [33, 44, 49, 52, 53]. Clinical experience with icodextrin, a colloid osmotic agent, is limited and, consequently, it should be used with caution in infants due to possible hyponatremia and a risk of rebound hypoglycaemia in the daytime [54, 55]. There is no evidence for the beneficial use of amino acid solutions in this age group [48].

### Recommendation 8: The most important measure of dialysis adequacy is appropriate growth and development. Frequent adjustments of the dialysis prescription and medications are necessary to control biochemical abnormalities and fluid status (1C).

Adequate dialysis should aim to achieve good electrolyte and acid–base control, appropriate fluid status and acceptable small solute clearance (residual and peritoneal). The most sensitive measure of appropriate dialysis in infants is appropriate growth and development [56]. Catch-up growth was recently described in half of the infants on chronic PD, and a positive relation was found between the length standard deviation score (SDS) and both fill volume and treatment duration [14]. The infant will need frequent assessment of both growth and psychomotor development and regular adjustments in dialysis prescriptions [57]. In the young infant these procedures are often associated with a prolonged period of initial hospitalisation. The successful management of infants at home requires performance of PD and daily measurements of weight, blood pressure, urine output, ultrafiltration and volume of delivered nutrients and fluids. The sum of urine output and UF needs to balance the volume of fluids necessary to provide adequate nutrition.

The frequency of ambulatory visits is fortnightly and can be extended in older, well-growing infants to every 3–4 weeks. At each visit infants will require precise weight, height and head circumference measurements (anthropometric equipment), with documentation on a growth chart and assessment of fluid status. These measurements provide important information for differentiating overhydration from weight gain. The fluid status can be assessed by clinical judgement of the infant’s skin and mucous membranes, changes in blood pressure and inappropriate changes in weight. It is frequently difficult to differentiate between fluid overload and increases in body mass. Ultrasound vena cava inferior measurements and bioimpedance are useful non-invasive methods, and atrial natriuretic peptide (ANP) levels may be helpful in equivocal situations, but none are yet recommended for routine clinical use.

Chronic hypervolaemia is common in anuric infants and leads to hypertension and cardiac impairment. It may be present in polyuric infants and is associated with poor growth; it has also been reported to cause sight loss (ischaemic optic neuropathy). Laboratory assessments are necessary at each visit for frequent adjustments of phosphate binders and remaining medications.

The frequent exchanges, short dwell times and low fill volume performed in infants may lead to adequate urea but relatively poor creatinine and phosphate elimination, which appears as a characteristic discrepancy of high kt/V (>3) and low creatinine clearance (<50 ml/min/1.73 m^2^/week). The impact of this condition is not clear but can be accepted if the infant is biochemically stable and growth is adequate. Sodium levels may decrease to levels present in the dialysis fluids (132 mEq/L). Serum sodium levels correlate poorly with sodium status. Many infants will require sodium supplementation, especially those with salt-losing nephropathies and/or high ultrafiltration. Sodium supplements might be beneficial for growth.

Despite frequent exchanges and biocompatible fluids with a normal pH, some infants still require bicarbonate supplementation. Others demonstrate metabolic alkalosis and will require lower buffer content dialysate.

### Recommendation 9: Nutritional management is an essential part of the management of infants on PD. Enteral feedings will be necessary for most infants to meet the protein and caloric requirements necessary to achieve linear growth (1B).

One of the major clinical issues of infant dialysis is adequate nutrition for maintaining adequate growth [58]. Multiple factors contribute to growth retardation in infants with CKD, including severe anorexia, vomiting, gastro-oesophageal reflux, fluid restriction, acidosis, salt-wasting, renal osteodystrophy, anaemia, alteration in the gut hormonal balance, altered peristalsis and psychosocial deprivation. Spontaneous intake of both nutrients and medications in the infant with CKD is rare, and oral supplementation and/or enteral feedings via nasogastric tubes or gastrostomy and supplemental feedings will be necessary for most infants to achieve linear growth [59]. Improvement of growth should not be taken for granted: a significant increase in weight-for-age and body mass index (BMI)-for-age, but not of height-for-age has been recently reported in children with CKD after gastrostomy tube feeding, and 50 % of tube-fed subjects were overweight or obese at the most recent evaluation [60]. Although recombinant human growth hormone (rhGH) has not been adequately investigated in infants, recent studies suggest that early GH treatment may improve growth [61]. A randomised controlled study has shown a good response of infants to rhGH without adverse effects despite concerns that infants on high caloric intake and PD may develop insulin resistance and glucose intolerance [62]. These preliminary studies suggest that a minority of infants who do not grow well despite good nutrition and adequate metabolic and clinical control may benefit from GH treatment with a significant improvement in body length. The advances made in intensive nutritional treatment, which have been achieved by the team work of nephrologists, surgeons, gastro-enterologists, dieticians, psychologists and dedicated cooperative parents, have dramatically improved the growth and development of infants on dialysis. Nevertheless, the psychological pressure of achieving adequate growth and the additional workload of intensive nutrition are viewed by some parents as a greater burden than performing PD.

An additional advantage of enteral feedings is the possibility of delivering the numerous oral medications prescribed, irrespective of their volume and taste and without the cooperation of the infant (sodium chloride, bicarbonate, phosphate binders, folic acid and ferrum sulphate, K-binding resins, antihypertensive drugs, vitamins and antibiotics).

Nutritional support includes both supplemental feedings and tube feedings via nasogastric tubes or gastrostomies. Tube feedings can be delivered by syringe as a daily supplementation of each natural feeding or by feeding pumps as nightly supplementation. The basic nutrients used for tube feeding are breast milk, infant formulas, specialised infant formulas for CKD and high calorie or high protein supplements. Nasogastric tubes may be distressing to the parents as they are visible, require exchange every 3–6 weeks, increase the risk of gastro-oesophageal reflux and may lead to food aspiration. They are not recommended for infants with existing gastro-oesophageal reflux [63–67]. The timing of gastrostomy tube placement is very important. When the infant’s degree of renal failure allows time for planning, a gastrostomy tube should be placed before the need for dialysis arises. When a child urgently requires dialysis, a gastrostomy may be placed at the same time as a PD catheter [24]. It is crucial to remember that gastrostomy tube placement in a child established on PD carries a very high risk of infections, including fungal peritonitis and loss of the peritoneal membrane [66, 67]. However, in the situation that a child already on PD develops growth failure and requires tube feeding, an open gastrostomy (Stamm procedure) with adequate peri-operative antibiotic and anti-fungal cover may be considered.

It is particularly important to place the gastrostomy tube as soon as early signs of poor weight gain are detected as more advanced growth failure and protein-energy wasting will lead to poor healing post-operatively.

Nissen fundoplication may be necessary if persistent vomiting is present despite optimisatio of anti-reflux medications. Non-infectious complications include leaks and oesophageal herniae.

The nutrient requirements are calculated individually for each infant, and the calorific and protein requirements differ by centre. Calories are calculated according to the required nutritional intake (RNI) for chronological age or height age. Glucose absorption from the dialysate can be significant and supply 10–20 kcal/kg/day. Most infants require RNI protein intake, which needs to be supplemented by protein dialysate loss and daily nitrogen losses to achieve a positive nitrogen balance [63, 64, 68]. Albumin loss in the ultrafiltrate is estimated to be 0.3 mg albumin/kg/day, but exact protein losses can be easily calculated from their concentration in total daily ultrafiltrate.

There is little information on the requirements for water-soluble vitamins, including folic acid. It would be reasonable to give the RNI for vitamins and micronutrients as for normal infants. In adults on PD, blood concentrations of some water-soluble vitamins C, B6 and folic acid are reported to be low. In children, supplements of these vitamins have been given, with the result that blood concentrations have met or exceeded normal values [63, 69].

### Recommendation 10: Centre-specific treatment is advocated for infectious complications of infant PD according to centre specific protocols (1B).

Mortality rates are fourfold higher in infants than in children aged >12 years and highest for those starting dialysis at <1 month of age [2–10, 14]. The major causes of death in children who initiate dialysis in infancy are infections, although dialysis-related complications as a cause of death are relatively rare [2, 5, 57, 70]. Co-morbidities (pulmonary disease and/or hypoplasia, severe developmental delay, congenital heart disease, congenital nephrotic syndrome) are important risk factors [12–18].

The principle underlying the treatment of peritonitis infections is preservation of the peritoneal membrane—and not preservation of the catheter. Fungal peritonitis or *Staphylococcus aureus* peritonitis is particularly difficult to treat and requires prompt assessment of the response to treatment and catheter removal, if appropriate. Haemodialysis as a short-term “rescue’ therapy” is feasible in infants [31, 70].

Peritonitis rates are higher in the youngest age group, and Gram-negative organisms (“diaper peritonitis”) are more frequent in infants [71]. In agreement with the findings of Boehm, oligoanuria is an important risk factor for peritonitis [72]. Gram-negative peritonitis responds poorly to empirical treatment with ceftazidime and first generation cephalosporin/glycopeptides [odds ratio (OR) 3.61, 95 % confidence interval (CI) 1.73–7.54, *p* < 0.001]. A worse response has also been observed with intermittent dosing of ceftazidime (OR 6.65, 95 % CI 2.07–21.4, *p* < 0.005) [73, 74]. A centre tailored empirical treatment is advocated for infants as well as for older children with infectious complications of PD. The intraperitoneal dosage for APD is frequently unknown for many antibiotics, and an initial 6-hourly “continuous ambulatory peritoneal dialysis” (CAPD) dwell regime is adopted. Systemic administration of antibiotics may be necessary in a child who is systemically unwell or has septicaemia. When possible antibiotic concentrations should be measured to avoid both over- and under-dosing. A switch to antibiotics with the lowest toxicity should be performed once antibiotic sensitivity is known, especially if aminoglycosides have been used for empiric therapy.

Fungal infections are more common in children with gastrostomies, and anti-fungal prophylaxis with oral nystatin and topical nystatin ointment around the gastrostomy tube is advisable [67].

The very young patient on PD may be prone to developing hypogammaglobulinaemia, but the latter’s relation to the increased risk of severe infection is not straightforward, and antibody levels to immunisations are usually normal. There is no evidence for the routine use of prophylactic immunoglobulins in all infants, but it is prudent to monitor and consider replacement therapy if low immunoglobulin levels are found in this high-risk population for sepsis [75].

### Recommendation 11: Provision of psychosocial support and family teaching support should be an integral part of the continuing care of infants with ESRD (1C). 

The first year of life is critical for brain growth, and deficits will have a significant impact on neurodevelopment. Early reports described profound neurologic deficits in infants with ESRD. More recent reports, however, show that the developmental outcome for survivors of an infant dialysis programme is encouraging. Improved outcomes are associated with intensive nutrition, correction of anemia and elimination of aluminium as phosphate binders [76, 77]. Good school performance has been reported in more recent evaluations of infant onset ESRD [7, 9]. Provision of psychosocial support and family teaching support should be an integral part of the continuing care of infants and young children with CKD5 to compensate for frequent hospitalisation and future poor school attendance due to chronic illness which influences academic achievement [24].

### Recommendation 12: PD is part of an integrated care approach for infants with CKD5 in which early renal transplantation is the ultimate goal (1B).

Although PD is feasible for both the newborn and the infant, it is a heavy burden for the family and a great challenge for the multispecialist team involved in their care. Renal transplantation lessens the family burden of everyday dialysis and management and improves renal function. Transplantation is associated with significant cognitive and psychomotor improvement and accelerated growth [29]. The timing of renal transplantation is dependent on both the condition and preparation of the infant and the expertise of the transplantation surgeon. Referral to a transplant center experienced in the transplantation of infants may be warranted. The infant on dialysis should be adequately nourished and clinically stable, needs to complete a vaccination schedule and often requires urologic surgery in preparation for transplantation [16, 37, 38, 78]. Transplantation as soon as the child is clinically and surgically stable has been advocated by some centres [79, 80]. The current recommendation for an adequate weight for transplantation is approximately 10 kg. Those newborns and infants that survive for transplantation will have long-term outcomes similar to those of older recipients of renal grafts and in fact have a better long-term graft survival than teenagers [8, 10, 79–82].

## Summary

Initiation of long-term dialysis in the infant as well as the neonate is an acceptable option for both parents and clinical staff but remains a challenge for both. Technical advances in automated PD have lessened the burden of dialysis. Short-term outcome has improved with intensive nutrition and a decrease in complication rates. The long-term outlook has also improved with increasing survival rates for dialysis and kidney transplantation in small children. A number of dilemmas remain, including the management of the infant with CKD who has severe co-morbidities and those infants deprived of family support. Although final outcomes are still not well defined, the available data support a more optimistic prognosis to earlier reports for both infants and neonates embarking on the challenge of RRT.

## References

[1] van der Heijden B, Dijk P, Verrier-Jones K, Jager K, Briggs D (2004). Renal replacement therapy in children: data from 12 registries in Europe. Pediatr Nephrol.

[2] Carey W, Talley L, Sehring S, Jaskula J, Mathias R (2007). Outcomes of dialysis initiated during the neonatal period for treatment of end-stage renal disease: a North American Pediatric Renal Trials and Collaborative Studies special analysis. Pediatrics.

[3] van Stralen K, Tizard E, Verrina E, Schaefer F, Jager K (2010). Demographics of paediatric renal replacement therapy in Europe: 2007 annual report of the ESPN/ERA-EDTA registry European Society for Paediatric Nephrology/ European Renal Association-European Dialysis and Transplant Association (ESPN/ERA-EDTA) registry study group. Pediatr Nephrol.

[4] Verrina E, Zacchello G, Perfumo F, Edefonti A, Sorino P, Bassi S, Andreetta B, Cattarelli D, Capasso G, Consalvo G (1995). Clinical experience in the treatment of infants with chronic peritoneal dialysis. Adv Perit Dial.

[5] Coulthard MG, Crosier J (2002). Outcome of reaching end stage renal failure in children under 2 years of age. Arch Dis Child.

[6] Jander A, Nowicki M, Tkaczyk M, Makulska I, Zwolińska D, Latoszyńska J, Boguszewska-Baczkowska A, Grenda R, Bałasz-Chmielewska I, Zagozdzon I, Załuska-Leśniewska I, Zurowska A, Stefaniak E, Zachwieja J, Leszczyńska B, Roszkowska-Blaim M, Zachwieja K, Pietrzyk JA, Wierciński R, Zoch-Zwierz W, Stankiewicz R (2006). Chronic peritoneal dialysis in infants—preliminary results of a multicenter survey. Przegl Lek.

[7] Laakkonen H, Hölttä T, Lönnqvist T, Holmberg C, Rönnholm K (2008). Peritoneal dialysis in children under two years of age. Nephrol Dial Transplant.

[8] Rheault M, Rajapal J, Chavers B, Nevins T (2009). Outcomes of infants < 28 days old treated with peritoneal dialysis for end-stage renal disease. Pediatr Nephrol.

[9] Mekahli D, Shaw V, Ledermann SE, Rees L (2010). Long-term outcome of infants with severe chronic kidney disease. Clin J Am Soc Nephrol.

[10] Wedekin M, Ehrich JH, Offner G, Pape L (2010). Renal replacement therapy in infants with chronic renal failure in the first year of life. Clin J Am Soc Nephrol.

[11] Uhlig K, Macleod A, Craig J, Lau J, Levey AS, Levin A, MoistL SE, Walker R, Wanner C, Lameire N, Eknoyan G (2001). Grading evidence and recommendations for clinical practice guidelines in nephrology. A position statement from Kidney Disease: Improving Global Outcomes (KDIGO). Kidney Int.

[12] Wood E, Hand M, Briscoe D, Donaldson L, Yiu V, Harley F, Warady B, Ellis E (2001). Risk factors for mortality in infants and young children on dialysis. North American Pediatric Renal Transplant Cooperative Study. Am J Kidney Dis.

[13] Shroff R, Ledermann S (2009). Long-term outcome of chronic dialysis in children. Pediatr Nephrol.

[14] Vidal E, Edefonti A, Murer L, Gianoglio MS, Pecoraro C, Sorino P, Leozappa G, Lavoratti G, Ratsch I, Chimenz R, Verrina E (2012). Peritoneal dialysis in infants: the experience of the Italian Registry of Paediatric Chronic Dialysis Nephrol. Dial Transplant.

[15] Tong A, Lowe A, Sainsbury P, Craig JC (2010). Parental perspectives on caring for a child with chronic disease: an in-depth interview study. Child Care Health Dev.

[16] Rönnholm KA, Holmberg C (2006). Peritoneal dialysis in infants. Pediatr Nephrol.

[17] Beaunoyer M, Snehal M, Li L, Concepcion W, Salvatierra O, Sarwal M (2007). Optimizing outcomes for neonatal ARPKD. Pediatr Transplant.

[18] Wühl E, Kogan J, Zurowska A, Matejas V, Vandevoorde R, Aigner T, Wendler O, Lesniewska I, Bouvier R, Reis A, Weis J, Cochat P, Zenker M (2007). Neurodevelopmental deficits in Pierson (microcoria-congenital nephrosis) syndrome. Am J Med Genet.

[19] Burguet A, Abraham-Lerat L, Cholley F, Champion G, Bouissou F, André J (2002). Terminal and pre-terminal chronic renal insufficiency in newborns in French neonatal intensive care units: survey of the French pediatric nephrologic society of resuscitation and emergency. Arch Pediatr.

[20] Fauriel I, Moutel G, Duchange N, Montuclard L, Moutard ML, Cochat P, Hervé C (2005). Decision making concerning life-sustaining treatment in paediatric nephrology: professionals’ experiences and values. Nephrol Dial Transplant.

[21] Shooter M, Watson AR (2000). The ethics of withholding and withdrawing therapy in infants. Pediatr Nephrol.

[22] Cohen C (1987). Ethical and legal considerations in the care of the infants with end stage renal disease where parents elect conservative therapy. Pediatr Nephrol.

[23] Broniszczak D, Ismail H, Nachulewicz P, Szymczak M, Drewniak T, Markiewicz-Kijewska M, Kowalski A, Jobs K, Smirska E, Rubik J, Skobejko-Włodarska L, Gastoł P, Mikołajczyk A, Kalicinski P (2010). Kidney transplantation in children with bladder augmentation or ileal conduit diversion. Eur J Pediatr Surg.

[24] Watson A, Gartland C (2001). Guidelines by an ad hoc European committee for elective chronic peritoneal dialysis in pediatric patients. Perit Dial Int.

[25] Van Dyck M, Sidler S, Proesmans W (1998). Chronic renal failure in infants: effect of strict conservative treatment on growth. Eur J Ped.

[26] González C, Bitsori M, Tullus K (2007). Progression of chronic renal failure in children with dysplastic kidneys. Pediatr Nephrol.

[27] Ismaili K, Schurmans T, Wissing KM, Hall M, Van Aelst C, Janssen F (2001). Early prognostic factors of infants with chronic renal failure caused by renal dysplasia. Pediatr Nephrol.

[28] Van Biesen W, Vanholder R, Veys N, Lameire N (2002). Peritoneal dialysis in anuric patients: concerns and cautions. Semin Dial.

[29] Rees L (2009). Long-term outcome after renal transplantation in childhood. Pediatr Nephrol.

[30] Shroff R, Wright E, Ledermann S, Hutchisson C, Rees L (2003). Chronic hemodialysis in infants and children under 2 years of age. Pediatr Nephrol.

[31] Paul A, Fraser N, Manoharan S, Williams AR, Shenoy M (2011). The challenge of maintaining dialysis lines in the under twos. J Pediatr Urol.

[32] Fischbach M, Edefonti A, Schröder C, Watson A, European Pediatric Dialysis Working Group (2005). Hemodialysis in children: general practical guidelines. Pediatr Nephrol.

[33] Fischbach M, Stefanidis C, Watson A, European Pediatric Peritoneal Dialysis Working Group (2002). Guidelines on peritoneal dialysis prescription in children. Nephrol Dial Transplant.

[34] Nüsken E, Dittrich K, Carbon R, Dötsch J (2010). Considering laparoscopic salvage options—is pre-emptive omentectomy necessary in paediatric peritoneal patients?. Klin Padiatr.

[35] Warchol S, Ziolkowska H, Roszkowska-Blaim M (2003). Exit-site infection in children on peritoneal dialysis: comparison of two types of peritoneal catheters. Perit Dial Int.

[36] Sojo E, Grosman M, Monteverde M, Bailez M, Delgado N (2004). Fibrin glue is useful in preventing early dialysate leakage in children on chronic peritoneal dialysis. Perit Dial Int.

[37] Alam S, Sheldon C (2008). Urological issues in pediatric renal transplantation. Curr Opin Urol.

[38] Nahas W, David-Neto E (2009). Strategies to treat children with end-stage renal dysfunction and severe lower urinary tract anomalies for receiving a kidney transplant. Pediatr Transplant.

[39] Fischbach M, Lahlou A, Eyer D, Desprez P, Geisert J (1996). Peritoneal dialysis prescription for neonates. Perit Dial Int.

[40] Fischbach M, Terzic J, Laugel V, Escande B, Dangelser C, Helmstetter A (2003). Measurement of hydrostatic intraperitoneal pressure : a useful tool for the improvement of dialysis prescription. Pediatr Nephrol.

[41] Fischbach M, Terzic J, Menouer S, Haraldsson B (2000). Optimal volume prescription for children on peritoneal dialysis. Perit Dial Int.

[42] Fischbach M, Haraldsson B, Helms P, Danner S, Laugel V, Terzic J (2003). The peritoneal membrane a dynamic dialysis membrane in children. Adv Perit Dial.

[43] Fischbach M, Terzic J, Chauvé S, Laugel V, Muller A, Haraldsson B (2004). Effect of peritoneal dialysis fluid composition on peritoneal area available for exchange in children. Nephrol Dial Transplant.

[44] Schröder C (2004). Optimal peritoneal dialysis : choice of volume and solution. Nephrol Dial Transplant.

[45] Verrina E, Cappelli V, Perfumo F (2009). Selection of modalities, prescription, and technical issues in children on peritoneal dialysis. Pediatr Nephrol.

[46] Fischbach M, Warady B (2009). Peritoneal dialysis prescription in children: bedside principles for optimal practice. Pediatr Nephrol.

[47] Schmitt C, Borzych D, Nau B, Wühl E, Zurowska A, Schaefer F (2009). Dialytic phosphate removal: a modifiable measure of dialysis efficacy in automated peritoneal dialysis. Perit Dial Int.

[48] Canepa A, Verrina E, Perfumo F (2008). Use of new peritoneal dialysis solutions in children. Kidney Int Suppl.

[49] Schmitt CP, Bakkaloglu SA, Klaus G, Schröder C, Fischbach M (2011). Solutions for peritoneal dialysis in children: recommendations by the European Pediatric Dialysis Working Group. Pediatr Nephrol.

[50] Haas S, Schmitt CP, Arbeiter K, Bonzel KE, Fischbach M, John U, Pieper AK, Schaub TP, Passlick-Deetjen J, Mehls O, Schaefer F (2003). Improved acidosis correction and recovery of mesothelial cell mass with neutral-pH bicarbonate dialysis solution among children undergoing automated peritoneal dialysis. J Am Soc Nephrol.

[51] Schmitt CP, von Heyl D, Rieger S, Arbeiter K, Bonzel K, Fischbach M, Misselwitz J, Pieper A, Schaefer F, Mid European Pediatric Peritoneal Dialysis Study Group (MEPPS) (2007). Reduced systemic advanced glycation end products in children receiving peritoneal dialysis with low glucose degradation product content. Nephrol Dial Transplant.

[52] Borzych D, Rees L, Ha IS, Chua A, Valles PG, Lipka M, Zambrano P, Ahlenstiel T, Bakkaloglu SA, Spizzirri AP, Lopez L, Ozaltin F, Printza N, Hari P, Klaus G, Bak M, Vogel A, Ariceta G, Yap HK, Warady BA, Schaefer F, International Pediatric PD Network (IPPN) (2010). The bone and mineral disorder of children undergoing chronic peritoneal dialysis. Kidney Int.

[53] Klaus G (2006). Prevention and treatment of renal osteodystrophy in children on chronic renal failure: European guidelines. Pediatr Nephrol.

[54] Michallat AC, Dheu C, Loichot C, Danner S, Fischbach M (2005). Long daytime exchange in children on continuous cycling peritoneal dialysis: preservation of drained volume because of icodextrin use. Adv Perit Dial.

[55] Dart A, Feber J, Wong H, Filler G (2005). Icodextrin reabsorption varies with age in children on automated peritoneal dialysis. Pediatr Nephrol.

[56] Coleman JE, Edefonti A, Watson AR (2001). Guidelines by an ad hoc committee on the assessment of growth and nutritional status in children on chronic peritoneal dialysis. Perit Dial Int.

[57] Rees L (2007). Long-term peritoneal dialysis in infants. Perit Dial Int.

[58] Rees L, Azocar M, Borzych D, Watson AR, Büscher A, Edefonti A, Bilge I, Askenazi D, Leozappa G, Gonzales C, van Hoeck K, Secker D, Zurowska A, Rönnholm K, Bouts AH, Stewart H, Ariceta G, Ranchin B, Warady BA, Schaefer F, International Pediatric Peritoneal Dialysis Network (IPPN) registry (2011). Growth in very young children undergoing chronic peritoneal dialysis. J Am Soc Nephrol.

[59] Watson AR (2006). Gastrostomy feeding in children on chronic peritoneal dialysis. Perit Dial Int.

[60] Sienna J, Saqan R, The J, Frieling M, Secker D, Cornelius V, Geary D (2010). Body size in children with chronic kidney disease after gastrostomy tube feeding. Pediatr Nephrol.

[61] Mencarelli F, Kiepe D, Leozappa G, Stringini G, Cappa M, Emma F (2009). Growth hormone treatment started in the first year of life in infants with chronic renal failure. Pediatr Nephrol.

[62] Santos F, Moreno ML, Neto A, Ariceta G, Vara J, Alonso A, Bueno A, Afonso AC, Correia AJ, Muley R, Barrios V, Gómez C, Argente J (2010). Improvement in growth after 1 year of growth hormone therapy in well-nourished infants with growth retardation secondary to chronic renal failure: results of a multicenter, controlled, randomized, open clinical trial. Clin J Am Soc Nephrol.

[63] Rees L, Shaw V (2007). Nutrition in children with CRF and on dialysis. Pediatr Nephrol 22:1689–1702 Erratum in. Pediatr Nephrol.

[64] Paglialonga F, Edefonti A (2009). Nutrition assessment and management in children on peritoneal dialysis. Pediatr Nephrol.

[65] KDOQI Work Group (2009). KDOQI clinical practice guideline for nutrition in children with chronic kidney disease: 2008 update. Am J Kidney Dis.

[66] Ledermann S, Spitz L, Moloney J, Rees L, Trompeter R (2002). Gastrostomy feeding in infants and children on peritoneal dialysis. Pediatr Nephrol.

[67] von Schnakenburg C, Feneberg R, Plank C, Zimmering M, Arbeiter K, Bald M, Fehrenbach H, Griebel M, Licht C, Konrad M, Timmermann K, Kemper M (2006). Percutaneous endoscopic gastrostomy in children on peritoneal dialysis. Perit Dial Int.

[68] Quan A, Baum M (1996). Protein losses in children on continuous cycler peritoneal dialysis. Pediatr Nephrol.

[69] Shaw V, Coleman J (2003). Nutritional management of renal disease in childhood. Ann Nestle.

[70] Shroff R, Rees L, Trompeter R, Hutchinson C, Ledermann S (2006). Long-term outcome of chronic dialysis in children. Pediatr Nephrol.

[71] Zurowska A, Feneberg R, Warady BA, Zimmering M, Monteverde M, Testa S, Calyskan S, Drozdz D, Salusky I, Kemper MJ, Ekim M, Verrina E, Misselwitz J, Schaefer F (2008). Gram-negative peritonitis in children undergoing long-term peritoneal dialysis. Am J Kidney Dis.

[72] Boehm M, Vecsei A, Aufricht C, Mueller T, Csaicsich D, Arbeiter K (2005). Risk factors for peritonitis in pediatric peritoneal dialysis: a single-center study. Pediatr Nephrol.

[73] Warady BA, Feneberg R, Verrina E, Flynn JT, Müller-Wiefel DE, Besbas N, Zurowska A, Aksu N, Fischbach M, Sojo E, Donmez O, Sever L, Sirin A, Alexander SR, Schaefer F (2007). Peritonitis in children who receive long-term peritoneal dialysis: a prospective evaluation of therapeutic guidelines. J Am Soc Nephrol.

[74] Li P, Szeto C, Piraino B, Bernardini J, Figueiredo A, Gupta A, Johnson D, Kuijper E, Lye W, Schaefer F, Struijk D (2010). Peritoneal dialysis related infections recommendations : 2010 update. Perit Dial Int.

[75] Neu AM, Warady BA, Lederman HM, Furth SL, Fivush BA (1998). Hypogammaglobulinemia in infants and young children maintained on peritoneal dialysis. Pediatric Dialysis Study Consortium. Perit Dial Int.

[76] Warady B, Belden B (1999). Kohaut E (1999) Neurodevelopmental outcome of children initiating peritoneal dialysis in early infancy. Pediatr Nephrol.

[77] Madden S, Ledermann S, Guerrero-Blanco M, Bruce M, Trompeter R (2003). Cognitive and psychosocial outcome of infants dialysed in infancy. Child Care Health Dev.

[78] Neuhaus T (2004). Immunization in children with chronic renal failure: a practical approach. Pediatr Nephrol.

[79] Humar A, Arrazola L, Mauer M, Matas A, Najarian J (2001). Kidney transplantation in young children: should there be a minimum age. Pediatr Nephrol.

[80] Chavers B, Najarian J, Humar A (2007). Kidney transplantation in infants and small children. Pediatr Transpl.

[81] Kari J, Romagnoli J, Duffy P, Fernando O, Rees L, Trompeter R (1999). Renal transplantation in children under 5 years of age. Pediatr Nephrol.

[82] Moudgil A, Martz K, Stablein DM, Puliyanda D (2011). Good outcome of kidney transplants in recipients of young donors: a NAPRTCS data analysis. Pediatr Transplant.

